# Structural Characterization by NMR of a Double Phosphorylated Chimeric Peptide Vaccine for Treatment of Alzheimer’s Disease

**DOI:** 10.3390/molecules18054929

**Published:** 2013-04-26

**Authors:** Karla Ramírez-Gualito, Monique Richter, Manolis Matzapetakis, David Singer, Stefan Berger

**Affiliations:** 1 Institute of Analytical Chemistry, University Leipzig, Johannisallee 29, Leipzig 04103, Germany; E-Mail: stberger@rz.uni-leipzig.de; 2 Institute of Bioanalytical Chemistry, University Leipzig, Deutscher Platz 5, Leipzig 04103, Germany; E-Mails: monique.richter@bbz.uni-leipzig.de (M.R.); david.singer@arcor.de (D.S.); 3 Instituto de Tecnologia Química e Biológica, Universidade Nova de Lisboa. Av. da República, Oeiras 2780-157, Portugal; E-Mail: matzman@itqb.unl.pt

**Keywords:** NMR spectroscopy, Biological Magnetic Resonance Data Bank, Alzheimer’s disease, peptide vaccine, B cell epitope, T cell epitope, Tau protein, hyperphosphorylation, *Mycobacterium tuberculosis*

## Abstract

Rational design of peptide vaccines becomes important for the treatment of some diseases such as Alzheimer’s disease (AD) and related disorders. In this study, as part of a larger effort to explore correlations of structure and activity, we attempt to characterize the doubly phosphorylated chimeric peptide vaccine targeting a hyperphosphorylated epitope of the Tau protein. The 28-mer linear chimeric peptide consists of the double phosphorylated B cell epitope Tau_229-237_[pThr231/pSer235] and the immunomodulatory T cell epitope Ag85B_241-255_ originating from the well-known antigen Ag85B of the *Mycobacterium tuberculosis*, linked by a four amino acid sequence -GPSL-. NMR chemical shift analysis of our construct demonstrated that the synthesized peptide is essentially unfolded with a tendency to form a β-turn due to the linker. In conclusion, the -GPSL- unit presumably connects the two parts of the vaccine without transferring any structural information from one part to the other. Therefore, the double phosphorylated epitope of the Tau peptide is flexible and accessible.

## 1. Introduction

The conformation of a peptide or protein can be relevant to its stability and function. Various intramolecular interactions are important for the relationship between the primary and tertiary structure. Structure determination of small peptides is a challenging task but necessary to obtain insights into their ability to bind to certain receptors; e.g., major histocompatibility complex (MHC) involved in immune response. To understand the relation between structure and function of peptides, it is necessary to consider folding initiation sites such as β-hairpin motifs [1] as well as α-helical and β-sheet like structures. It should also be taken into account that medium-range peptides commonly exist in solution as complex mixtures of conformers of similar energies and correlation times. This inherent inhomogeneity often renders them difficult to study and determine specific interactions through the space associated with certain tertiary structural elements [2]. Nevertheless, recent methodologies are able to uncover evidence of small populations of folded structures in such seemingly unfolded ensembles in water [3,4]. Intrinsically disordered proteins (IDPs) have been shown to be functional, despite their lack of well-defined structure, imposing a new perspective on the relationship between primary protein sequence and function and necessitating the development of an entirely new set of experimental and analytical techniques [5,6]. Analysis of chemical shifts, by identifying the deviations of the chemical shifts of certain nuclei from random coil value, is one useful tool for detecting secondary structure elements and has been commonly used in the characterization of proteins by NMR [7,8,9,10]. With this, NMR analysis becomes a useful tool for the study of local conformational preferences that encode biological functions [11,12,13].

β-turns are the most common type of non-repetitive structures recognized in proteins and are important for providing a directional change within the polypeptide chain [14]. There is also much evidence that β-turns are the key structures for molecular recognition and protein folding [15]. Since they were first recognized, much effort has been made for their analysis and for prediction of their presence from a specific amino acid sequence [16].

The rational design of peptide based vaccines has become an important target for the treatment of infectious diseases, cancer and neurodegenerative diseases like the Alzheimer’s disease (AD) [17,18,19]. AD is the most common form of dementia that is placing a considerable and increasing burden on patients, caregivers and the society. AD is clinically characterized by a progression from episodic memory problems to a global decline of cognitive function including the inability to acquire or recall memories, motor dysfunctions and personality changes [20]. The impairment of cognitive function in patients suffering from AD is accompanied by two pathological protein aggregates found in their brain. These aggregates are commonly referred as senile plaques (SP) and neurofibrillary tangles (NFT’s). SP are extracellular deposits made up by the β-amyloid peptide whereas NFT’s mainly consist of hyperphosphorylated Tau protein, which aggregates within neurons [21]. The biochemical cause of the disease still remains to be understood but there is an agreement that reducing the plaque and tangle burden e.g., by immunotherapy, is beneficial for the patients [22,23]. Due to severe difficulties in AD related immunization trials, alternatives, such as the rational design of anti-Aβ and anti-phospho Tau peptide vaccines, have become very important [24,25].

The 28-mer chimeric AD-specific peptide vaccine reported in this study is composed of the doubly phosphorylated Tau B cell epitope Tau_229-237_[pThr231/pSer235] and an immunomodulatory T cell epitope originating from the antigen Ag85B of the *Mycobacterium tuberculosis*. In our construct, both epitopes have been linearly connected via a four amino acid linker, which was previously utilized for the design of such short peptide vaccines [26,27,28]. The aim of the present study was to associate a potential correlation between the immunological properties of the vaccine and its conformation. We report the measurement and analysis of the NMR chemical shifts of ^1^H, ^13^C and ^15^N to study the conformational state of the 28-mer peptide vaccine and its artificial loop.

## 2. Results and Discussion

**Figure 1:**
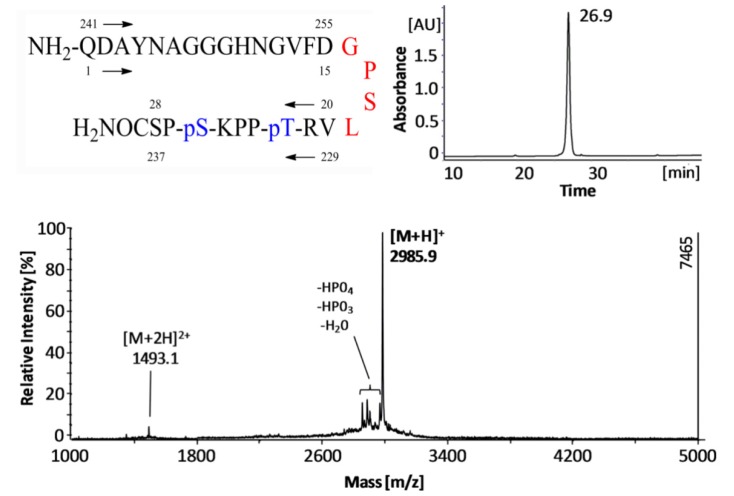
Amino acid sequence of the AD-specific peptide vaccine Ag85B_241-255_-GPSL-Tau_229-237_[pThr231/pSer235] (upper left panel). The Tau sequence is numbered according to the longest human isoform with 441 amino acids. For convenience the peptide was renumbered from 1 to 28 from N- to C-terminus. RP-HPLC chromatogram (upper right panel) and MALDI mass spectrum (lower panel) of the purified Ag85B_241-255_-GPSL-Tau_229-237_[pThr231/pSer235].

The peptide vaccine (Figure 1) comprised of 28 amino acids can be divided in three sections, the immunomodulatory T cell epitope Ag85B_241-255_ from *Mycobacterium tuberculosis,* the four amino acid linker sequence -GPSL-, and the B cell epitope Tau_229-237_[pThr231/pSer235]. The double phosphorylated Tau epitope is thought to be AD specific and a promising target for immunotherapy [24,29]. The peptide was synthesized by standard Fmoc-chemistry and was post-synthetically phosphorylated using phosphoramidite. The purity was above 95% confirmed by analytical HPLC and MALDI mass spectrometry (Figure 1).

**Figure 2:**
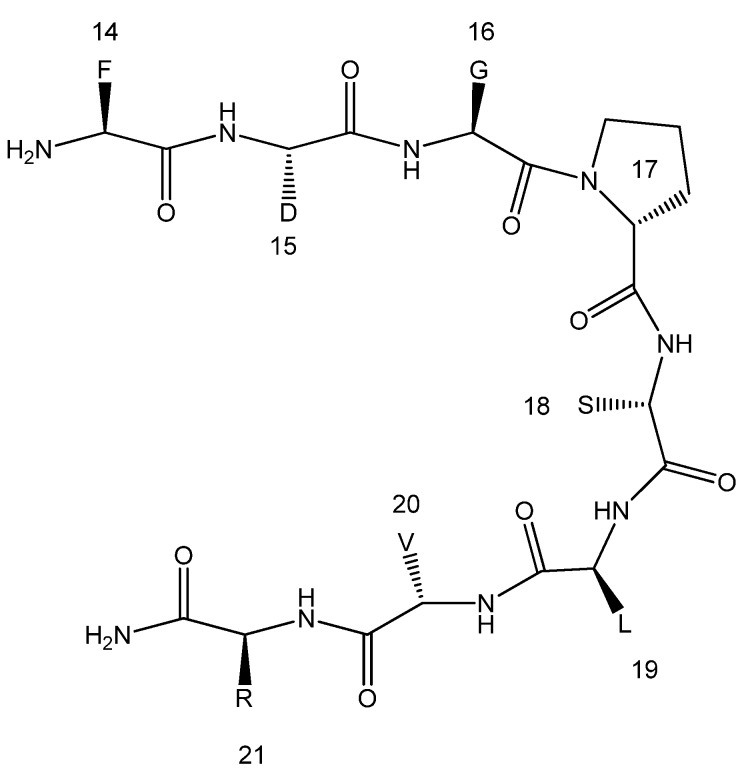
Schematic diagram of the loop formed by the residues Gly16-Leu18.

The spectra collected were analyzed using the CCPNmr software [30] and we were able to complete 85.7% of the type-specific assignment of 24 out of the 28 spins systems involved. Gln1, Asp2, Pro27 and Ser28, could not be observed, likely due to increased flexibility and exchange with the bulk solvent in these parts of the molecule that rendered the specific resonances of these residues invisible to NMR. The assignment for 85.0% of the hydrogen atoms, 66.9% of the carbon atoms and 64.9% of the nitrogen atoms was made. Hydrogen assignment corresponds to 64.2% assigned hydrogen atoms of the backbone and 83.9% of the side chain. Analysis of the NOESY spectrum revealed only short range NOEs between neighboring residues (i, i + 1 and i, i + 2) but no long range ones. 35 NOEs of various types of (i, i + 1) and 7 of (i, i + 2) ranges were identified. More specifically dγN (i, i + 2) were identified between Asp15 and Val13, dγα (i, i + 2) from Val20 to Thr22, dδN (i, i + 2) between Arg21 and Leu19, dβN (i, i + 2) from Arg21 to Pro23, dγN (i, i + 2) from Thr22 to Val20, dαβ (i, i + 2) between Pro23 and Arg21 and finally dβN (i, i + 2) from Lys25 to Pro23. These NOEs are found in the regions bracketing the Gly16, Pro17, Ser18 and Leu19, GPSL hypothesized linker suggesting a specific spatial order of the peptide. However, the absence of longer range NOEs implies an overall conformational inhomogeneity of the construct. We assume that the artificial linker (Figure 2) is forcing both arms of the peptide chain into a more limited conformational space while not significantly affecting the overall flexibility of the active epitopes, as is also evident from the very small chemical shift deviations from the random coil of those positions [31]. Efforts to study the conformational exchange by variations of temperature were limited by the H_2_O/D_2_O working temperature range and by the solubility of the peptide so it was not possible to reach conclusions by temperature dependent NMR studies. In addition, the possibility of our observations originating from spin diffusion is not significant since the mixing times used for NOESY (Nuclear Overhauser Effect SpectroscopY) are too small to cause spin diffusion.

The GPSL linker, has been previously used to stabilize other peptidic structures, e.g., α-helical structures of such short peptide vaccines. Additionally, it was used as a flexible connection for peptides, which adopt their tertiary structure independently [26,27,28]. In the loop the residues glycine and proline potentiate a β-turn in the polypeptide, whereas the side-chain of serine will favor hydrogen bonds with the free H^N^ of the backbone. The side chain of leucine in the sequence is important for hydrophobic interactions [26].

**Figure 3:**
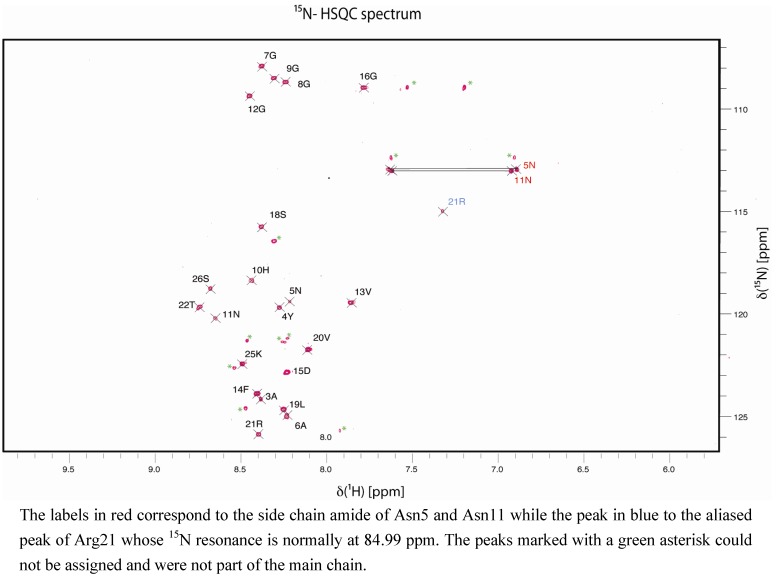
^15^N-HSQC spectrum of the AD-specific peptide vaccine (Ag85B_241-255_-GPSL-Tau_229-237_[pThr231/pSer235]).

Due to the lack of long range NOEs in our system and in order to better probe its structural features, we attempted to also compare the chemical shift differences of several atoms of the amino acids of the peptide sample with random coil chemical shifts, to identify any significant deviations statistically. The chemical shifts of our system are reported in the Supplementary Information for the ^1^H, ^13^C and ^15^N atoms (Table S1) and are deposited in BMRB (BMRB ID = 19112) [32]. In the ^15^N-HSQC (Heteronuclear Single Quantum Coherence) spectrum (Figure 3) a group of four glycines (Gly6, Gly8, Gly9 and Gly12) was identified, the H^N^ had ^1^H and ^15^N chemical shifts between 8.2 to 8.5 ppm and between 107 to 109 ppm, respectively. In contrast, the H^N^ of Gly16 had a ^1^H chemical shift of 7.8 ppm suggesting this amino acid experienced a different environment compared to the remaining glycines in the peptide, implying a potential function within the GPSL loop. We excluded the possibility of aggregation being a factor since we did not observe any chemical shift dependence in measurements made on samples of concentrations different by an order of magnitude. In addition, diffusion measurements did not suggest aggregation.

**Figure 4:**
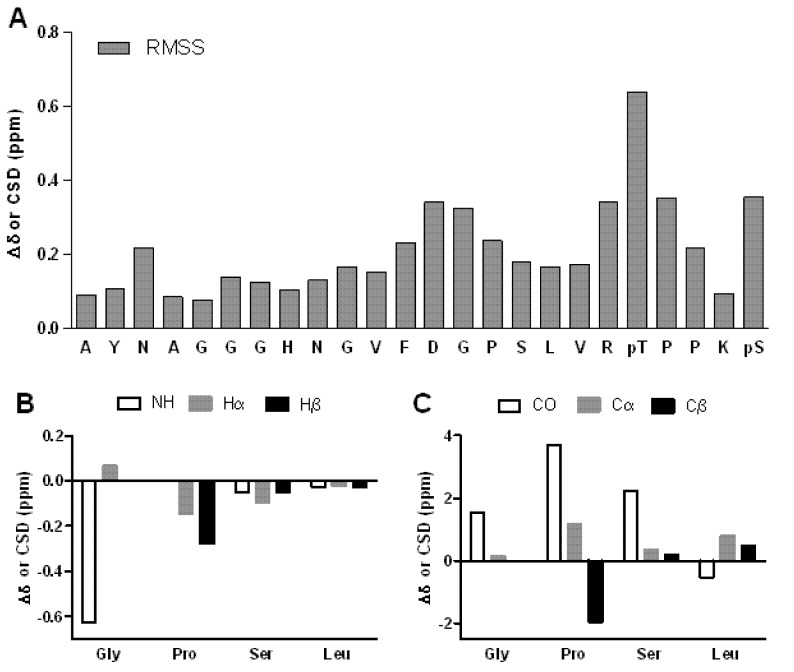
(**A**) Graphical representation of the chemical shift differences versus random coil chemical shifts of the 28-mer peptide calculated through the RMSS. (**B**) Chemical shift differences for H^N^, Hα and Hβ. (**C**) Chemical shift differences for CO, Cα and Cβ. **B** and **C** were calculated considering only the artificial loop region chemical shifts and random coil values.

In addition to the above example of the Gly16, a wider statistical analysis was performed in order to quantify the chemical shift information of the complete amino acid sequence. The protons most sensitive to conformational changes associated with secondary structure are the Hα, which have been widely used to study the conformation of peptides and for the determination of structural elements in proteins and peptides folding studies [26,33,34,35]. In addition, the chemical shifts of Cα and Cβ have a significant correlation with dihedral angles and are often used for the structural analysis of peptides and proteins [36]. Moreover H^N^ have been used as probes of a consistent pattern for β-hairpins and three-stranded β sheets [7]. Since the number of identified or available chemical shifts for each amino acid was not the same, we opted to use a combined analysis of the differences with the use of the RMSS (Root of the Mean Sum of the Squares) value. This analysis yields a single value for each amino acid representing its cumulative deviation from random coil behavior and due to the use of weighted averages it is independent of the number of chemical shifts it is composed of. The results of the statistical analysis (Figure 4A) showed that some populations deviate from the random coil values in agreement with the presence of a partial secondary structure present in the 28-mer peptide. The most important changes were observed in the region from Phe14 to Val20. Also, the chemical shifts revealed a significant difference in the region comprising the GPSL sequence. Another important feature was observed for pThr22 and pSer26, the two phosphorylated amino acids, which have chemical shifts that differ from the non-phosphorylated ones. Interestingly, amino acids Phe14 and Asp15 showed a difference from the random coil chemical shifts, but this may be attributed to the ring current effect of the Phe aromatic ring over Asp15.

A specific analysis was done using the chemical shift difference of the loop sequence GPSL. The chemical shift difference (Δδ) was defined as Δδ = δ_exp_ − δ_rc_, where δ_exp_ belongs to the experimental chemical shift and δ_rc_ to the random coil shift. Several studies support the argument that Δδ could be indicative for the identification of α-helices, β-sheets and loops, but such an analysis depends on the definition of the random coil state for a given amino acid sequence which is not easy to reach [35,37,38]. The random coil state has been defined as the state where the dihedral angles of each residue is independent of the conformation of the neighboring residues, but some effects such as solvation and ring current effects will often influence local chemical shifts [39]. The results for the chemical shift differences for the atoms H^N^, Hα and Hβ (Figure 4B) are consistent with the corresponding results for chemical shift analysis. As previously mentioned, the most significant difference was observed for the H^N^ atom of Gly16, supporting its special role in the GPSL loop. In the case of the C atoms (Figure 4C), the results are also consistent displaying significant differences for Gly16, Pro17 and Ser18. Only for Leu19 it was not possible to observe any significant shift differences for all protons and C atoms.

**Figure 5:**
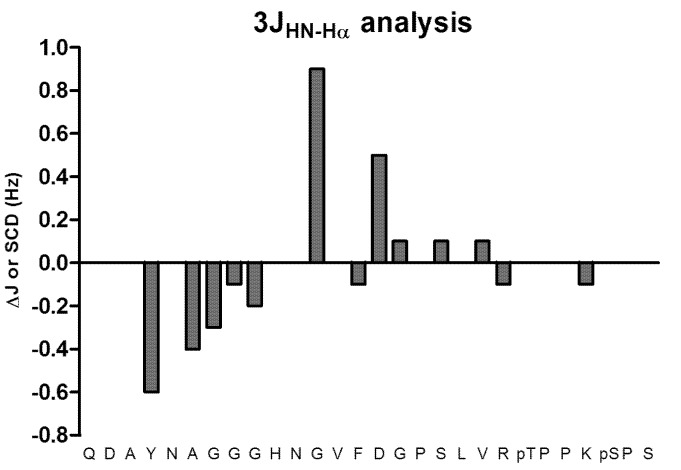
Graphical representation of the scalar coupling differences (Δ*J*) of the experimental data from those calculated by Flexible-Meccano (Δ*J* = *J*_Exp_ − *J*_FM_).

^3^*J*_HN-Hα_ coupling constant carries structural information and has a long recognized relation with the ϕ dihedral angle of the peptide plane. That relation is described by the parameterized Karplus relationship [40]. We were able to extract these values from the analysis of the DQF-COSY (Double Quantum Filter-COrrelationSpectroscopY). However, the evaluation of these Js turned to be difficult in the absence of an estimate of the expected values for the random coil of our peptide. For this purpose we used, the Flexible-Meccano program to calculate 100,000 random structures of our designed peptide that were not in violation of the Ramachandran space and subsequently calculate the average Js over all of the calculated structures using the Karplus relationship. We were then able to compare these values with those extracted from the COSY and the results of the differences between these two sets of values in shown in Figure 5 (Table S2). Only negligible difference between the experimental and the theoretical values can be observed suggesting that the ^3^*J*_HN-Hα_ couplings are less sensitive than chemical shift for the detection of structural features.

## 3. Experimental

### 3.1. Peptide Synthesis

The phosphorylated Ag85B241-255-GPSL-Tau229-237[pThr231/pSer235] peptide was synthesized by standard Fmoc/*t*-butyl chemistry using a polystyrene-based Rink Amide (MBHA) resin (0.65 mmol/g, MultiSynTech GmbH, Witten, Germany). Fmoc-protected amino acids (0.5 mol/L, MultiSynTec GmbH) were activated with diisopropylcarbodiimide in the presence of 1-hydroxybenzotriazole (DIC/HOBt) and coupled automatically in eight molar excess by the Syro2000 multiple peptide synthesizer (MultiSynTech GmbH). Amino acids to be phosphorylated were incorporated with unprotected side chains, *i.e.*, Fmoc-Ser-OH and Fmoc-Thr-OH. Global postsynthetic phosphorylation was done as described elsewhere [23]. In brief, free hydroxyl groups were phosphitylated with 10 equivalents of dibenzyl-*N,N*-diisopropyl-phosphoramidite in the presence of 20 equivalents of 1*H*-tetrazole in acetonitrile/dimethylformamide at room temperature for 90 min and 16 h. After washing, phosphitylated amino acids were oxidized with 100 eq *tert*-butyl hydroperoxide (*t*-BuOOH) in decane at room temperature for 90 min. After synthesis, the peptidyl resin was washed with dimethylformamide and methylene chloride, air dried and the peptide was cleaved with 5% water, 4% thioanisol, 4% *m*-cresol, and 2% ethanedithiol in trifluoroacetic acid (TFA) at room temperature for 2 h. Peptides were precipitated with ice cold diethyl ether, washed three times, and air dried. The peptide was purified on an Äkta HPLC System (Amersham Bioscience GmbH, Freiburg, Germany) using a Jupiter C18-column (21.2 mm × 250 mm, Phenomenex Inc., Torrance, CA, USA). Elution was performed by a linear gradient with an increase of 1% acetonitrile per minute in the presence of 0.1% TFA (10 mL/min, 220 nm). The purity of the peptide was confirmed by analytical RP-HPLC using a Jupiter C18 column (2.0 × 150 mm, 3 µm, 300 Å) and the correct masses confirmed by matrix-assisted laser desorption/ionization time-of-flight mass spectrometry (MALDI-TOF-MS; 4700 proteomic analyzer; Applied Biosystems GmbH, Darmstadt, Germany) operated in positive ion-mode using α-cyano-4-hydroxy-cinnamic acid (BrukerDaltonics GmbH, Bremen, Germany). Alternatively, peptide purity was confirmed by LC-ESI-MS (Qstar pulsar, ESI QqTOF Hybrid mass spectrometer, Applied Biosystems) using acetonitrile/water as eluents in the presence of 0.1% formic acid. The peptide was obtained in a yield of 14%.

### 3.2. NMR Spectroscopy

The NMR sample were prepared by dissolving the peptide in 500 µL phosphate buffer (Na_2_HPO_4_/NaH_2_PO_4_) at pH 5.8 with a 9:1 H_2_O:D_2_O or D_2_O ratio. The Sørensen phosphate buffer at pH 5.8 was prepared with 92 mL of a solution 0.2 M NaH_2_PO_4_ and 8 mL of a 0.2 M solution of Na_2_HPO_4_ using D_2_O as a solvent for both cases [41,42]. Solution pH values were verified using a pH electrode (Spintrode, Hamilton, Bonaduz, Switzerland) inside the NMR tube. Spectra were externally referenced to the singlet resonance of 4,4-dimethyl-4-silapentane-1-sulfonic acid (DSS) at 0 ppm. Spectra were recorded on a Bruker AVANCE-700 with a cryoprobe and an AVANCE-III 800. All the spectra were recorded using a sample with a concentration of 1.6 mM at 300 K. The experiments used were the, TOtal Correlation SpectroscopY (TOCSY), COrrelation SpectroscopY (COSY), Nuclear Overhauser Effect SpectroscopY (NOESY), Rotating frame Overhauser Effect SpectroscopY (ROESY), Heteronuclear Multiple Bond Correlation (HMBC), Heteronuclear Single Quantum Coherence (^15^N-HSQC) Heteronuclear Multiple Quantum Coherence (^13^C-HMQC), and Double Quantum Filter-COrrelation SpectroscopY (DQF-COSY). Excitation sculpting sequence was used for water suppression. The mixing times of NOESY experiments were set to150–250 ms.

### 3.3. Statistical Analysis

The statistical analysis of the combined chemical shift deviations from random coil values was achieved by calculating RMSS (root of the mean of sum squares) of all available nuclei, namely H^N^, N, Hα, Hβ, Cα and Cβ involved for each amino acid except for glycines. The formula involves the use of correction factors calculated from the Biological Magnetic Resonance Data Bank (BMRB) statistics [43,44] representing the average variances of the nuclei in relation with the gyromagnetic constant of each nuclei. The values considered for the analysis are the experimental chemical shift (δX*exp)* and the random coil shift (δX*rc)* while *n* represents the number of terms used in this equation.

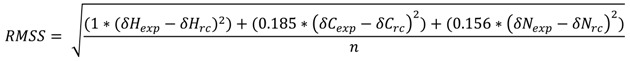


The random coil values used were obtained from Schwarzinger *et al*. [45]. The value of the RMSS gives a collective estimate of how far each amino acid is from the random coil, and it is not affected for the number of nuclei considered in each case.

The weighting factors used in the RMSS formula were obtained from the standard deviation of the respective values reported in the diamagnetic set of chemical shifts of the BMRB. These statistical deviations originated from comparable number of measurements.

### 3.4. ^3^J_HN-Hα_ Couplings Analysis

^3^*J*_HN-Hα_ couplings constants values were obtained from the DQF-COSY spectrum, by measuring the splitting of the antiphase peaks. The digital resolution of the spectra used was 1.8Hz/point and the half height line width of the antiphase peaks ranged from 1.8–2.5Hz which is less than two times the scalar couplings measured, limiting the experimental error to below 1% [46]. This analysis uses an algorithm to build multiple, different copies of the same polypeptide chain by randomly sampling amino acid-speciﬁc backbone dihedral angle {φ/ψ} potential wells. The peptide chain is constructed by using the selected {φ/ψ} pairs to sequentially connect peptide planes. The calculation of the scalar ^3^*J*_HN-Hα_ considers the use of the following Karplus relationship to calculate the values for each conformer:

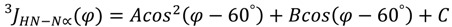

where *A* = 6.4, *B* = −1.4, *C* = 1.9 and θ = ϕ − 60° [47,48].

## 4. Conclusions

We have shown, despite the fluxional behavior of a small peptide a careful chemical shift analysis, using available data bank values, can identify structural features efficiently. The current NMR analysis of the Alzheimer’s disease related peptides vaccines Ag85B_241-255_-GPSL-Tau_229-237_[pThr231/pSer235] demonstrated the potential presence of a β-turn comprising the linker sequence GPSL but no significant tertiary structure of the peptide vaccines. The conformation of the GPSL β-turn is not stable, but contributes to the overall structure and was therefore detectable. The GPSL sequence acted as a flexible linker joining the B cell and the T cell epitope, of the vaccine without transporting structural information to the other part. Contrary to this study, a local polyproline type II helix was previously reported for the phosphorylated Tau sequence [49] while the predicted structure of the tuberculosis T cell epitope is partially α-helical [50]. However, our results agree with the observation that proline is an amino acid which promotes flexible structures [31]. The existence of i, i+2 NOEs in the region of the loop, is evidence of local organization and smaller conformational inhomogeneity than the rest of the molecule. These longer range NOEs can only be obtained if the amides involved retain their relative positions in the ms time scale which is not possible in a random coil environment. This tendency of preorganization is not observed in the rest of the molecule, and is in agreement with the presence of the loop. The efficacy of the vaccine to induce an immune response directed towards the double phosphorylation site, the specificity of the phosphorylation dependent antibodies and the role of the T cell epitope is subject of ongoing work and might result in a novel immunization strategy for the treatment of Alzheimer’s disease. Preliminary data suggest that this first generation antigenic peptide has immunogenic properties despite the absence of significant structure. Future iteration of this peptide design will explore different structural elements and the combined results will allow us to make the correlations of structure and antigenic activity.

## Supplementary Materials

^1^H-700 MHz spectrum of the phosphorylated peptide (Figure S1), TOtal Correlation SpectroscopY (TOCSY) spectrum (Figure S2). Chemical shift assignment of the AD-specific peptide vaccine Ag85B_241-255_-GPSL-Tau_229-237_[pThr231/pSer235] (Table S1), ^3^*J*_NH-Hα_ analysis from experimental data (Exp) and Flexible Meccano (FM) ensemble (Table S2).

Supplementary materials can be accessed at: http://www.mdpi.com/1420-3049/18/5/4929/s1.
