# Supplementary material for: Structural Characterization by NMR of a Double Phosphorylated Chimeric Peptide Vaccine for Treatment of Alzheimer’s Disease
Source: Molecules. 2013 Apr 26;18(5):4929–41. doi: 10.3390/molecules18054929 (PMC6269680; doi:10.3390/molecules18054929)
Supplement: Supplementary file 1 [file molecules-18-04929-s001.pdf]

Supplementary Information

Figure S1. ^{1}H-700 MHz spectrum of the AD-specific peptide vaccine Ag85B$_{241\text{-}255}$-GPSL-Tau$_{229\text{-}237}$[pThr231/pSer235].

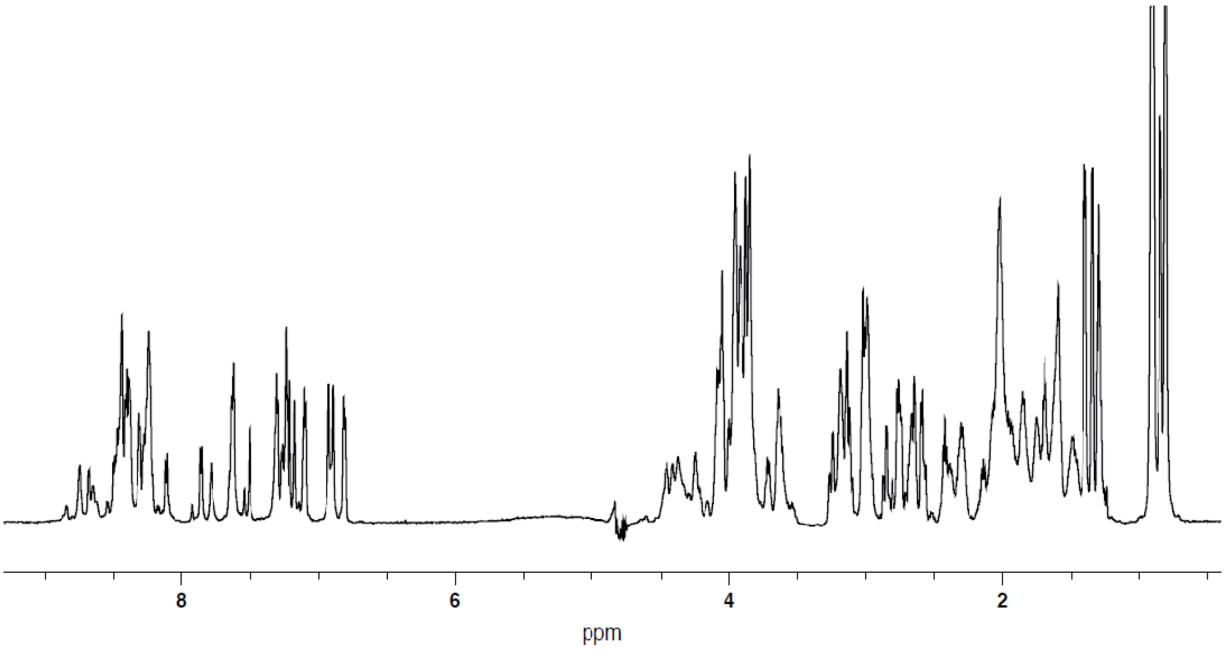

Figure S2. TOtal Correlation SpectroscopY (TOCSY) spectrum of the AD-specific peptide vaccine Ag85B$_{241\text{-}255}$-GPSL-Tau$_{229\text{-}237}$[pThr231/pSer235].

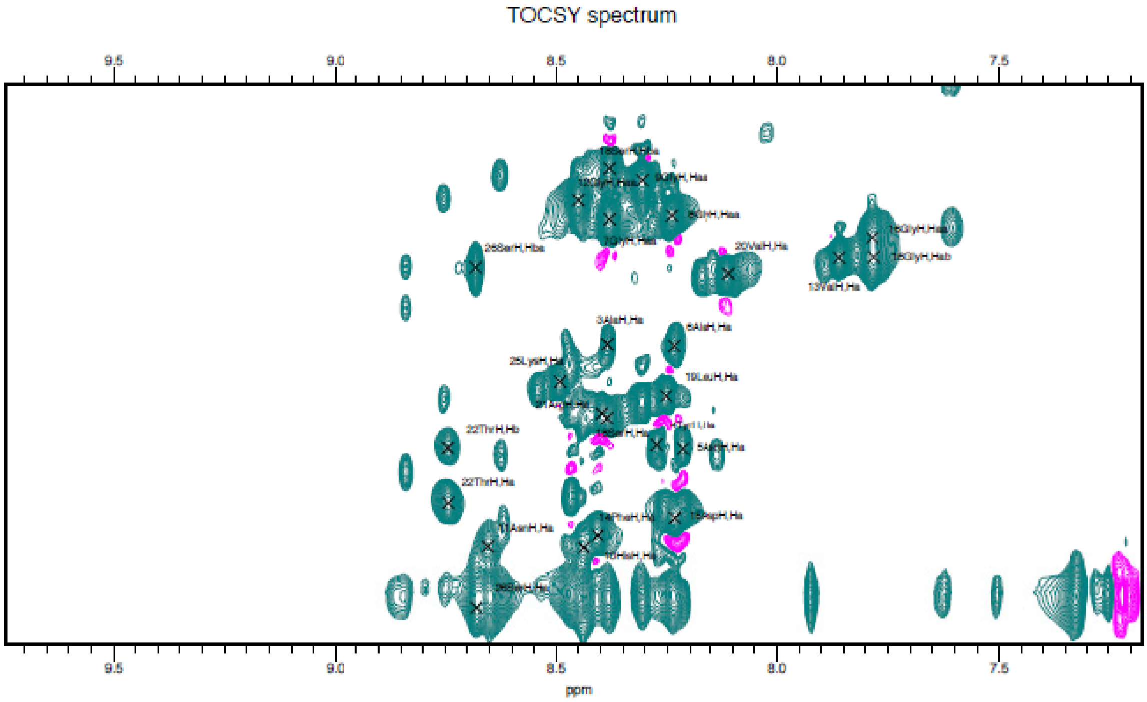

Table S1. Chemical shift assignment of the AD-specific peptide vaccine $Ag85B_{241-255}$-GPSL-$Tau_{229-237}$[pThr231/pSer235].

Amino acid	Atom name	Atom type	Value
3Ala	CO	13C	178.613
3Ala	Cβ	13C	19.793
3Ala	H	1H	8.384
3Ala	Hα	1H	4.244
3Ala	Hβ*	1H	1.285
3Ala	N	15N	124.155
4Tyr	CO	13C	176.250
4Tyr	Cα	13C	58.820
4Tyr	Cβ	13C	39.167
4Tyr	Cδ	13C	133.706
4Tyr	Cε	13C	118.808
4Tyr	Cγ	13C	131.280
4Tyr	H	1H	8.274
4Tyr	Hα	1H	4.475
4Tyr	Hβa	1H	2.989
4Tyr	Hδ*	1H	7.101
4Tyr	Hε*	1H	6.810
4Tyr	N	15N	119.694
5Asn	CO	13C	176.004
5Asn	H	1H	8.216
5Asn	Hα	1H	4.480
5Asn	Hβa	1H	2.650
5Asn	Hβb	1H	2.758
5Asn	Hδ2a	1H	6.891
5Asn	Hδ2b	1H	7.644
5Asn	N	15N	119.398
5Asn	Nδ2	15N	112.956
6Ala	CO	13C	178.522
6Ala	Cα	13C	53.427
6Ala	Cβ	13C	19.668
6Ala	H	1H	8.234
6Ala	Hα	1H	4.246
6Ala	Hβ*	1H	1.396
6Ala	N	15N	124.996
7Gly	Cα	13C	46.057
7Gly	H	1H	8.380
7Gly	Hαa	1H	3.963
7Gly	N	15N	107.905
8Gly	Cα	13C	45.873
8Gly	H	1H	8.239
8Gly	Hαa	1H	3.953
8Gly	N	15N	108.691
9Gly	Cα	13C	45.816
9Gly	H	1H	8.307
9Gly	Hαa	1H	3.876

Table S1. *Cont.*

Amino acid	Atom name	Atom type	Value
9Gly	N	15N	108.495
10His	Cα	13C	56.004
10His	Cβ	13C	29.800
10His	Cδ2	13C	120.563
10His	Cε1	13C	137.120
10His	Cγ	13C	132.426
10His	H	1H	8.438
10His	Hα	1H	4.702
10His	Hβa	1H	3.117
10His	Hβb	1H	3.248
10His	Hδ2	1H	7.211
10His	Hε1	1H	8.426
10His	N	15N	118.373
11Asn	Cα	13C	53.992
11Asn	Cβ	13C	39.498
11Asn	H	1H	8.651
11Asn	Hα	1H	4.703
11Asn	Hβa	1H	2.754
11Asn	Hβb	1H	2.861
11Asn	Hδ2a	1H	6.922
11Asn	Hδ2b	1H	7.619
11Asn	N	15N	120.193
11Asn	Nδ2	15N	113.019
12Gly	Cα	13C	45.988
12Gly	H	1H	8.450
12Gly	Hαa	1H	3.918
12Gly	N	15N	109.375
13Val	Cα	13C	62.965
13Val	Cβ	13C	33.271
13Val	Cγa	13C	21.356
13Val	H	1H	7.858
13Val	Hα	1H	4.049
13Val	Hβ	1H	1.963
13Val	Hγa*	1H	0.807
13Val	N	15N	119.458
14Phe	Cα	13C	58.112
14Phe	Cβ	13C	40.304
14Phe	Cδ*	13C	132.464
14Phe	Cε*	13C	131.936
14Phe	Cγ	13C	139.659
14Phe	Cξ	13C	130.393
14Phe	H	1H	8.406
14Phe	Hα	1H	4.679
14Phe	Hβa	1H	2.966
14Phe	Hβb	1H	3.155
14Phe	Hδ*	1H	7.236

Table S1. *Cont.*

Amino acid	Atom name	Atom type	Value
14Phe	Hε*	1H	7.304
14Phe	Hξ	1H	7.265
14Phe	N	15N	123.887
15Asp	CO	13C	176.355
15Asp	Cα	13C	54.609
15Asp	H	1H	8.233
15Asp	Hα	1H	4.644
15Asp	Hβa	1H	2.650
15Asp	Hβb	1H	2.745
15Asp	N	15N	122.855
16Gly	H	1H	7.784
16Gly	Hαa	1H	4.025
16Gly	CO	13C	176.434
16Gly	Cα	13C	45.536
16Gly	N	15N	108.966
17Pro	Hα	1H	4.454
17Pro	Hβa	1H	1.927
17Pro	Hβb	1H	2.295
17Pro	Hδa	1H	3.629
17Pro	Hδb	1H	3.642
17Pro	Hγa	1H	2.024
17Pro	Hγb	1H	2.298
17Pro	Cα	13C	64.187
17Pro	Cβ	13C	32.836
17Pro	Cδ	13C	50.547
17Pro	Cγ	13C	28.038
18Ser	H	1H	8.381
18Ser	Hα	1H	4.412
18Ser	Hβ	1H	3.849
18Ser	CO	13C	177.637
18Ser	Cα	13C	59.021
18Ser	Cβ	13C	64.260
18Ser	N	15N	115.746
19Leu	H	1H	8.252
19Leu	Hα	1H	4.360
19Leu	Hβ	1H	1.592
19Leu	Hδa*	1H	0.848
19Leu	Hδb*	1H	0.900
19Leu	Hγ	1H	1.605
19Leu	CO	13C	177.656
19Leu	Cα	13C	56.261
19Leu	Cβ	13C	42.934
19Leu	Cδa	13C	24.186
19Leu	Cδb	13C	25.475
19Leu	Cγ	13C	27.646
19Leu	N	15N	124.652
20Val	CO	13C	176.294

Table S1. *Cont.*

Amino acid	Atom name	Atom type	Value
20Val	Cα	13C	62.763
20Val	Cβ	13C	33.209
20Val	Cγa	13C	21.650
20Val	H	1H	8.112
20Val	Hα	1H	4.088
20Val	Hβ	1H	2.030
20Val	Hγa*	1H	0.912
20Val	N	15N	121.738
21Arg	CO	13C	175.057
21Arg	Cα	13C	56.388
21Arg	Cβ	13C	31.716
21Arg	Cγ	13C	27.512
21Arg	H	1H	8.396
21Arg	Hα	1H	4.401
21Arg	Hβa	1H	1.751
21Arg	Hβb	1H	1.854
21Arg	Hδa	1H	3.187
21Arg	Hε	1H	7.325
21Arg	Hγa	1H	1.622
21Arg	Hξ*	1H	1.293
21Arg	N	15N	125.856
21Arg	Ne	15N	84.990
22Thr	Cα	13C	60.572
22Thr	Cβ	13C	73.766
22Thr	Cγ2	13C	21.244
22Thr	H	1H	8.743
22Thr	Hα	1H	4.605
22Thr	Hβ	1H	4.476
22Thr	Hγ2*	1H	1.340
22Thr	N	15N	119.675
23Pro	Cα	13C	62.347
23Pro	Cβ	13C	31.583
23Pro	Cδ	13C	31.796
23Pro	Cγ	13C	28.197
23Pro	Hα	1H	4.658
23Pro	Hβa	1H	1.861
23Pro	Hβb	1H	2.378
23Pro	Hδa	1H	3.715
23Pro	Hδb	1H	3.945
23Pro	Hγa	1H	1.997
23Pro	Hγb	1H	2.083
24Pro	Cα	13C	63.616
24Pro	Cβ	13C	33.050
24Pro	Cδ	13C	51.030
24Pro	Cγ	13C	28.087
24Pro	Hα	1H	4.449
24Pro	Hβa	1H	1.907

Table S1. *Cont.*

Amino acid	Atom name	Atom type	Value
24Pro	Hβb	1H	2.298
24Pro	Hδa	1H	3.637
24Pro	Hδb	1H	3.810
24Pro	Hγa	1H	2.023
25Lys	CO	13C	177.060
25Lys	Cα	13C	56.646
25Lys	Cβ	13C	33.851
25Lys	Cδ	13C	29.606
25Lys	Cε	13C	42.772
25Lys	Cγ	13C	25.117
25Lys	H	1H	8.491
25Lys	Hα	1H	4.326
25Lys	Hβa	1H	1.758
25Lys	Hβb	1H	1.831
25Lys	Hδa	1H	1.699
25Lys	Hεa	1H	3.020
25Lys	Hγa	1H	1.487
25Lys	N	15N	122.431
26Ser	CO	13C	173.601
26Ser	Cβ	13C	66.117
26Ser	H	1H	8.679
26Ser	Hα	1H	4.834
26Ser	Hβa	1H	4.070
26Ser	N	15N	118.769

Table S2. $^3J_{HN\text{-}H\alpha}$ analysis from experimental data (Exp) and Flexible Meccano (FM) ensemble.

Amino acid	$^3J_{HN\text{-}H\alpha}$ Exp (Hz) [a]	$^3J_{HN\text{-}H\alpha}$ FM (Hz)	$\Delta J = J_{Exp} - J_{FM}$
1Gln		7.1	
2Asp			
3Ala		6.3	
4Tyr	7.0	7.6	-0.6
5Asn		7.5	
6Ala	6.0	6.3	-0.4
7Gly	5.9	6.2	-0.3
8Gly	6.1	6.2	-0.1
9Gly	6.0	6.2	-0.2
10His		7.4	
11Asn		7.5	
12Gly	7.1	6.2	0.9
13Val	7.8	7.8	0.0
14Phe	7.5	7.5	-0.1
15Asp	7.7	7.2	0.5
16Gly	5.7	5.7	0.1
17Pro			
18Ser	7.0	6.9	0.1

Table S2. *Cont.*

**Amino acid**	**$^3J_{HN\text{-}H\alpha}$ Exp (Hz)** [a]	**$^3J_{HN\text{-}H\alpha}$ FM (Hz)**	**$\Delta J = J_{Exp} - J_{FM}$**
19Leu		7.3	
20Val	8.0	7.8	0.1
21Arg	7.1	7.2	-0.1
22Thr		7.8	
23Pro			
24Pro			
25Lys	7.0	7.2	-0.1
26Ser		7.8	
27Pro			
28Ser			

a. The digital resolution of DQF-COSY spectrum was 1.8 Hz/point.
